# PP13 Access Barriers After The Incorporation Of Technologies Into The Unified Health System In Brazil

**DOI:** 10.1017/S026646232510233X

**Published:** 2025-12-29

**Authors:** Vanessa Pirolo

## Abstract

**Introduction:**

Diabetes is a condition that affects public health based on its increased incidence, prevalence, morbidity, and mortality. According to the latest Atlas of the International Diabetes Federation, it is estimated that there are 537 million adults with the condition; for Brazil, the estimate is 16 million. Investment in diabetes and its complications exceeds USD42 billion in Brazil.

**Methods:**

This national study was conducted from 1 July to 22 August 2024. Interviews were conducted online with 1,843 Brazilians over 18 years of age with diabetes. The study’s objective was to identify barriers to diabetes treatment in Brazil after the incorporation of health technologies in the Unified Health System (SUS) aimed at diabetes. Data were processed according to the profile of reported diabetes diagnosis in the National Health Survey 2019, conducted by the Brazilian Institute of Geography and Statistics.

**Results:**

The preliminary study indicated that 56 percent of those diagnosed with diabetes are over 60 years of age, 58 percent have elementary education, 54 percent have a monthly family income of up to two minimum wages, 75 percent have type 2 diabetes, 67 percent say they have hypertension, and 63 percent have high cholesterol. Obesity was reported by 39 percent, 82 percent use oral medication, 67 percent undergo tests through the SUS, and 84 percent obtain free medication through the SUS. Of those interviewed, 60 percent cite the availability of medication as the main factor in monitoring the condition, followed by attention from doctors (53%).

**Conclusions:**

The aging population poses challenges for diabetes control in Brazil and will further strain the public health system. Current barriers, such as bottlenecks in care and lack of medicines and doctors, are likely to worsen. Vulnerable groups with lower levels of education and income are currently the most affected. With the increasing age of the population, the system will spend more resources and may have difficulty sustaining itself in a few years.

